# The Energy Computation Paradox and *ab initio* Protein Folding

**DOI:** 10.1371/journal.pone.0018868

**Published:** 2011-04-25

**Authors:** John C. Faver, Mark L. Benson, Xiao He, Benjamin P. Roberts, Bing Wang, Michael S. Marshall, C. David Sherrill, Kenneth M. Merz

**Affiliations:** 1 Quantum Theory Project, University of Florida, Gainesville, Florida, United States of America; 2 Center for Computational Molecular Science and Technology, School of Chemistry and Biochemistry, and School of Computational Science and Engineering, Georgia Institute of Technology, Atlanta, Georgia, United States of America; Massachusetts Institute of Technology, United States of America

## Abstract

The routine prediction of three-dimensional protein structure from sequence remains a challenge in computational biochemistry. It has been intuited that calculated energies from physics-based scoring functions are able to distinguish native from nonnative folds based on previous performance with small proteins and that conformational sampling is the fundamental bottleneck to successful folding. We demonstrate that as protein size increases, errors in the computed energies become a significant problem. We show, by using error probability density functions, that physics-based scores contain significant systematic and random errors relative to accurate reference energies. These errors propagate throughout an entire protein and distort its energy landscape to such an extent that modern scoring functions should have little chance of success in finding the free energy minima of large proteins. Nonetheless, by understanding errors in physics-based score functions, they can be reduced in a *post-hoc* manner, improving accuracy in energy computation and fold discrimination.

## Introduction

A widely studied and yet largely unsolved problem in computational biochemistry is the *ab initio* protein-folding problem – the prediction of three-dimensional protein structure from an amino acid sequence [1], [2]. In recent years physics-based methods (those that explicitly model inter- and intramolecular interactions of a chemical system), combined with extensive conformational searches and sampling, have been explored as a general solution to the problem. The basis of any physics-based method used to study protein folding is the thermodynamic hypothesis - that the biologically active (native) fold is a free energy minimum [3]. This is the most widely used paradigm, although there are a few known exceptions to the rule [4], [5]. Molecular dynamics (MD) simulations are commonly used to analyze the folding kinetics of a protein using physics-based potentials, however the timescales needed to fully simulate the folding processes of large proteins can be prohibitively long [6], [7], [8], [9]. Monte Carlo-based search and minimization techniques in conjunction with physics-based potentials are also employed [10]. Unfortunately, these and other physics-based methods have had difficulty in correctly predicting protein folds of chains longer than 100 amino acids [11], [12].

One proposed explanation for the failure of current methods of physics-based folding of large proteins is that “*the primary obstacle to de novo protein structure prediction is conformational sampling*” [13]. Indeed, the high number of degrees of freedom makes it difficult to find the free energy minimum of a large protein. However, based on evidence from folding kinetics, a protein's energy landscape may have some predictable features in its overall shape. Levinthal noted that even though large proteins have access to vast amounts of conformational space, they transition from denatured states to folded states surprisingly quickly, as if the protein only selectively samples the available conformational space [14]. Based on this, it has been inferred that a protein's energy landscape is shaped like a high-dimensional funnel with very many high-energy states surrounding a deep global minimum (native state) [15], [16]. Depending on the protein, this funnel can have smooth or jagged slopes with variable inclination, which determines the folding rate [17]. With this type of folding landscape, the protein can easily “fall into” (perhaps after overcoming local minima) the folded state from any of the unfolded states without having to sample the entire energy surface. If the funnel concept is an accurate model of protein folding energy landscapes, then exhaustive conformational sampling should not be necessary, but adequate sampling remains an important component of a folding algorithm due to the likely presence of local minima in the energy surface, especially for slower folding proteins.

**Figure 1:**
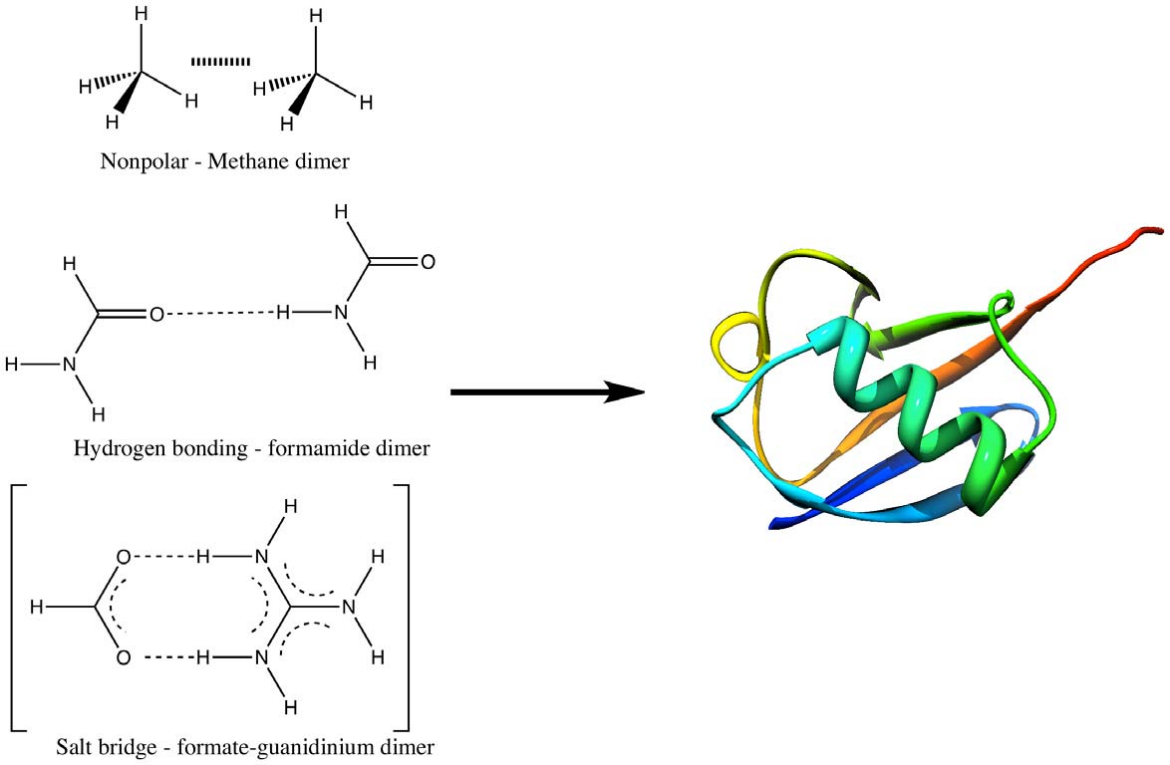
Example model systems used to build-up interactions in proteins. Accurate interaction energies for the model systems are assumed to yield accurate global interaction energies for a folded protein.

While sampling clearly plays a significant role in the ultimate solution to the *ab initio* protein folding problem, it is important not to overlook the role energy functions play. Physics-based *ab initio* protein folding attempts to calculate the relative free energies of protein conformations and energetically separate mis-folded structures from native ones. The typical foundation of physics-based potentials used in *ab initio* folding studies is the classical force field and its derivatives [18]. These are generally built in a piecewise manner based on model systems representing interactions found in proteins and are then extended to full protein systems. It has been assumed that the ability to accurately represent small model systems will yield an accurate representation of a full protein. That is, uncertainties in the energies of the model systems of ±1 kcal/mol are assumed to yield similar errors in the energy of the much larger protein systems (see Figure 1) [19]. Moreover, it is generally thought that because force fields are parameterized, they are largely subject to small random errors. Paradoxically we show that (1) although physics-based score functions yield small uncertainties for small model systems, these uncertainties increase dramatically with system size and that (2) most computational methods, even those that have been parameterized, contain large systematic and random errors when applied to macromolecules. Hence, we conclude that current energy functions introduce such significant uncertainties into physics-based folding exercises that improving accuracy in energy computation is just as important as sampling in solving the *ab initio* protein folding problem.

This point is clearly illustrated in recent studies of the Pin1 WW domain carried out by two groups using two force fields of similar construction, but with different parameter choices. Schulten and co-workers attempted to fold Pin1 using the CHARMM force field and long MD simulations (10 µs), but were unsuccessful [20]. This was later shown to be due to issues with the force field utilized [21]. However, recent long MD simulations (1 ms) of Shaw and co-workers succeeded in folding Pin1 using a modified AMBER force field (ff99sb) [22]. The forms of these two force fields both trace their roots from the consistent force field of Lifson [18], [23], [24] and co-workers and are similar in construction, but are parameterized differently. This comparison shows the range of uncertainty present in force fields, which can yield success or failure, but the origin of this has not been well understood until the present work.

The impact of our observation affects any method that attempts to compute the total interaction energies of any macromolecular process including: protein folding, protein-ligand docking, crystal isomorph prediction, assembly of nanomaterials, and others. In a previous study following our initial hypothesis [25], we applied statistical error analysis to the problem of protein-ligand docking [26]. In this study, it was observed that systematic and random errors do indeed quickly accumulate throughout large interacting chemical systems. For several of the score functions examined, the overall error estimates in the total interaction energy exceeded the experimental free energy of ligand binding. In the case of protein folding, error accumulation is also expected to be significant due to the high number of chemical interactions involved in a protein fold [19].

### Analysis of Error Propagation in Protein Folds

Errors inherent in a calculation or measurement can be described as either systematic or random. Systematic errors are predictable in both sign and magnitude, while random errors are not predictable in sign or magnitude. Because of this difference, systematic errors propagate as a simple sum, while random errors propagate as the square root of sum of squares [27]. Propagated systematic error is correctable, since it describes an overall predictable shift in the measured value. Propagated random errors are not easily correctable, and are measured and reported as the accumulation of error from all random error sources. Large random error is a characteristic of a very imprecise method of measurement. In searching the energy surface of a protein for the global free energy minimum, total error from all sources should be minimized.

**Figure 2:**
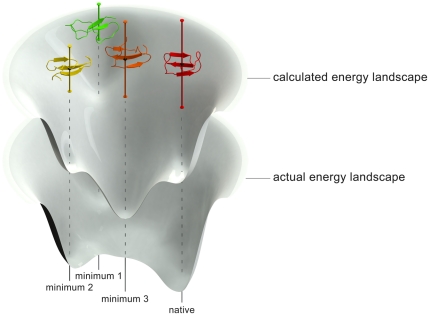
Distortions in computed energy landscapes due to error propagation. If each microstate of a protein under study contains a significant amount of error in its calculated energy (shown here as error bars), computed folding surfaces become distorted with respect to the actual folding surface. This effect introduces difficulty in distinguishing between local minima on the folding surface and in finding the native folds of proteins. This effect is magnified for especially large proteins with many intramolecular contacts contributing to their stable protein folds.

To illustrate the effects of error propagation on the protein-folding problem, imagine a protein's energy surface as a funnel surrounding the global minimum structure (folded state). In general these surfaces are not smooth, but often contain many local minima outside of the native state. If the folded protein has, for example, 100 independent chemical contacts (*e.g.* van der Waals interactions and hydrogen bonds between residues), and each is computationally modeled to chemical accuracy (i.e., within 1 kcal/mol random error with respect to an experimental measurement) [28], then random error propagation yields a total error of ±10 kcal/mol. This would imply that our hypothetical computational model would have difficulty in distinguishing the native state from any other state with overlapping error bars within 10 kcal/mol. Thus our computational model may correctly find several local minima, but if they differ in energy by less than the error bar magnitudes, the position of the global minimum (which is usually the native fold), could not be determined with much certainty (see Figure 2). Dill realized the issue of error propagation, leading him to suggest that a target of 0.1 kcal/mol *per amino acid* would be an acceptable level of error for a protein of 100 amino acids yielding an overall random error bar of only ±1 kcal/mol [19]. Given that each amino acid can have several intramolecular contacts, each with an associated error, achieving this level of accuracy is indeed a very challenging endeavor.

In our attempt to estimate and correct for errors in physics-based energy scores used in protein folding, we have taken an approach previously described by Merz [25]. Intramolecular interactions involved in protein folds are broken down into chemical fragments and associated with reference interaction energies obtained using converged quantum chemical calculations or experimental measurements, if available. Energies from more approximate theories are then compared to the reference interaction energies to form fragment-based error estimates. Estimating the error of a series of these fragment-based interactions contained within a protein fold and then propagating these errors throughout all fragments in the fold yields an estimate of the total error associated with a computed total energy for the protein. The error is broken down into a systematic portion which can be corrected for and a random portion which cannot, but can be reported as an error bar.

It is important to remember that the thermodynamic principle of protein folding applies to the free energy, not the interaction energy (differences of total electronic energies) that we use here. The total electronic energy of folding, ΔE_folding_, is just one part of the folding free energy, ΔG_folding_, obtained using the master equation for the computation of the folding free energy from a fully unfolded reference structure [19].
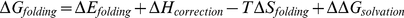
(1)Here ΔH_correction_ represents enthalpic corrections to the electronic energy, ΔS_folding_ is the entropy change of folding and ΔΔG_solvation_ is the difference in the solvation free energy of the folded and unfolded states.

In order to reliably calculate the free energies of native and nonnative protein folds, the errors associated with each term of Equation 1 must be minimized. In view of this, our interaction energy error estimates should be considered “best-case scenario” or lower-limit free energy error estimates because they neglect the error coming from the enthalpy, entropy, and solvation energy terms. Nonetheless, if the potential energy surface is not well modeled this will impact the quality of any estimate of entropy because a poor quality potential energy surface can have effects on entropy estimates that can be difficult to predict (*e.g.*, sampling of non-physical states). To obtain a reliable estimate of the free energy it is essential that ΔE be well reproduced to ensure the quality of the ΔS estimate. While it is true that systematic errors in the three remaining terms of Equation 1 may cancel favorably with systematic errors in ΔE, this effect has not yet been studied in detail. Random error estimates, however, will only increase with the addition of terms with nonzero uncertainties.

Our method of error analysis assumes that electronic interaction energies are additive, even though free energies of interacting fragments are not [19]. This approximation is supported by both statistical mechanics [29] and isothermal calorimetry experiments [30] involving protein-ligand interactions, but deviations from additivity will impact the overall quality of our error estimates. Nonetheless, it is very instructive to examine error estimates for the ΔE_folding_ term because, within our model, we can compare any physics-based score function to chemically accurate quantum chemical methods providing reliable estimates of the magnitudes of energy errors and their contribution to ΔG_folding_. If the ΔE_folding_ errors are small, their impact on the uncertainty in ΔG_folding_ will be small; otherwise they will have a significant impact on the accuracy of ΔG_folding_ and the ability to predict native protein folds.

## Methods

### Interacting Fragment Database Generation

In order to generate a reference database of fragment-based interacting systems involved in protein folds, we examined a native fold of ubiquitin (PDBID: 1UBQ) [31] in detail. After adding and optimizing hydrogens in AMBER [32] with the ff99sb force field [33], the structure was viewed using Chimera [34], which was used to highlight van der Waals contacts and hydrogen bonds resulting in a total of 42 of the former and 50 of the latter. Each resulting fragment interaction was evaluated in terms of gas-phase interaction energy using a number of different methods. In generating these fragments, hydrogen “link atoms” were used to replace the severed bonds with the remainder of the protein. Energies were evaluated with the ff99sb force field [33], the Generalized Amber Force Field (GAFF) [35], ff03 [36], AM1 [37], PM3 [38], PM6 [39], PDDG [40], PM6-DH2 [41], HF, MP2, B97-D [42], M06, and M06-L [43]. The ff99sb and ff03 force field methods underwent an atomic charge scaling procedure to produce correct net charges on the database fragments. This was necessary because the sum of parameterized force field charges on one fragment often did not equal the total charge used when calculating electronic energy with a QM reference method. Unless the force field charges are scaled properly, additional errors due to the lack of charge conservation are introduced [44].

The *ab initio* quantum-based methods employed several basis sets and included the counterpoise correction for basis set superposition error. Møller-Plesset perturbation theory through second order (MP2) with complete basis set extrapolations (CBS) [45] from the aug-cc-pVTZ and aug-cc-pVQZ basis sets (hereafter abbreviated as aXZ: X = D,T,Q) were used for most of the reference values. Based on previous reports [46] and our experience with error analysis on protein-ligand systems, the coupled cluster method with single, double, and perturbative triple excitations (CCSD(T)) CBS energies showed the largest improvement from MP2/CBS for systems containing aromatic groups. However, the present case contained no aromatic-aromatic interactions and only eight total aromatic-nonpolar contacts. Hence, CCSD(T)/CBS reference energies were computed (as described in our previous work [26]) only for these fragments. The addition of more protein molecules and specific interaction types [47] to our reference database would further improve our ability to estimate errors, but this is a time-consuming endeavor requiring a large number of high-level quantum mechanical energy calculations [48].

The ff99sb and GAFF calculations were conducted with the AMBER 11 suite of programs [32], and the ff03 energies were calculated with the Schrödinger package [49]. AM1, PM3, PM6, and PDDG energies were calculated with DivCon [50], and PM6-DH2 energies were calculated with MOPAC2009 [51]. HF, MP2, B97-D, M06, and M06-L energies were calculated with Gaussian 09 [52], and the CCSD(T)/CBS corrections used to generate reference values were calculated with Molpro 2009 [53] and NWChem 5.1 [54].

## Results

**Figure 3:**
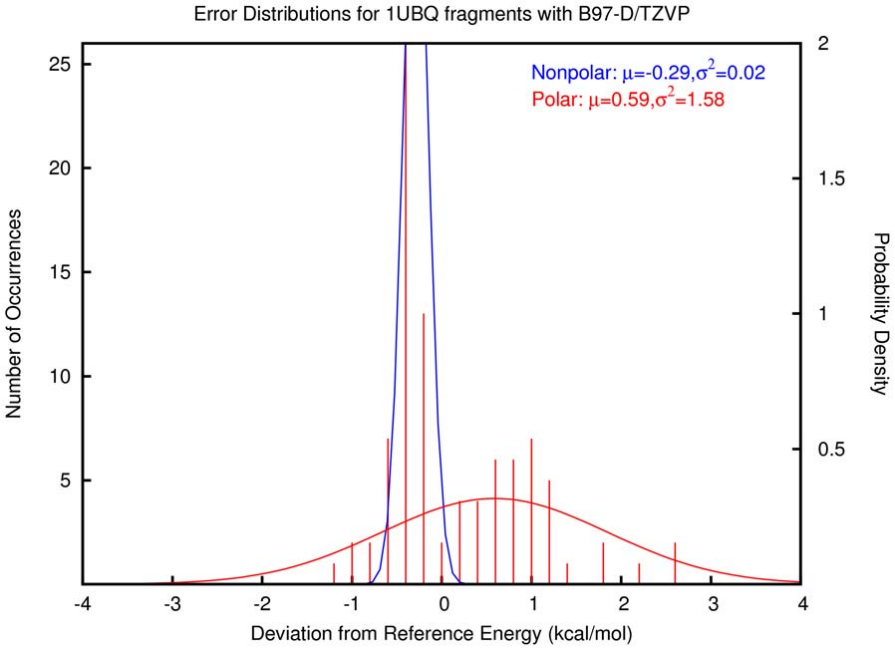
Histogram and probability density functions describing errors in B97-D/TZVP absolute electronic interaction energies of molecular fragments built from the native fold of ubiquitin.

A summary of the fragment-based interaction energy deviations from reference energies is displayed in Table 1. The absolute deviations from our reference data were fitted to Gaussian error probability density functions with parameters μ (mean error per interaction) and σ (standard deviation of the errors). The resulting plots are presented in Table S1. The error distribution for B97-D/TZVP is shown as an example in Figure 3. The fragments were divided into two classes: nonpolar (van der Waals - blue) and polar (hydrogen bonding - red) interactions. In the case of B97-D/TZVP, the mean error, representing the correctable systematic error, is −0.29 kcal/mol and 0.59 kcal/mol per interaction for nonpolar and polar interactions, respectively. The variance, representing random error, is 0.02 (kcal/mol)^2^ for nonpolar and 0.158 (kcal/mol)^2^ for polar interactions. Thus this computational model has a relatively precise description of van der Waals interactions with only a slight offset, but it has a wider distribution of errors for polar interactions.

**Table 1:** Interaction Energy Error Statistics of the 1UBQ Fragment Database.

Method	μ_All_	σ^2^_All_	μ_VdW_	σ^2^_VdW_	μ_Polar_	σ^2^_Polar_	R-factor^a^
GAFF	0.36	3.26	0.25	0.36	0.46	5.64	0.127
FF99SB^b^	0.73	4.04	0.12	1.27	1.22	5.83	0.170
FF03^b^	0.83	6.61	0.18	0.81	1.36	10.86	0.259
AM1	3.15	9.50	1.04	0.70	4.85	10.28	0.373
PM3	2.65	7.89	0.14	0.77	4.67	4.59	0.352
PM6	1.67	2.24	0.84	0.32	2.34	2.82	0.211
PM6-DH2	0.30	1.23	−0.09	0.10	0.62	1.95	0.071
PDDG	3.21	16.23	−0.62	0.90	6.30	7.48	0.484
HF/6-31G*	1.94	1.32	2.27	1.14	1.68	1.30	0.153
HF/aDZ	2.14	1.22	2.29	1.11	2.02	1.29	0.176
HF/aTZ	2.10	1.17	2.28	1.10	1.95	1.17	0.171
HF/aQZ	2.08	1.16	2.28	1.10	1.93	1.15	0.170
MP2/6-31G*	1.24	0.64	1.12	0.28	1.34	0.91	0.146
MP2/aDZ	0.48	0.16	0.21	0.01	0.69	0.19	0.061
MP2/aTZ	0.16	0.02	0.05	0.00	0.24	0.02	0.023
B97-D/TZVP	0.20	1.06	−0.29	0.02	0.60	1.58	0.087
M06/6-31G*	0.75	0.42	0.63	0.12	0.85	0.64	0.104
M06/aTZ	0.73	0.16	0.57	0.08	0.85	0.19	0.090
M06-L/6-31G*	0.71	0.43	0.40	0.10	0.96	0.57	0.103
M06-L/aTZ	0.75	0.14	0.55	0.07	0.91	0.14	0.096

Mean and variance of interaction energy deviations (kcal/mol) from reference energies (a mix of MP2/CBS and CCSD(T)/CBS) for the interacting fragment molecules present in ubiquitin. The set of fragments was divided into 42 van der Waals interactions and 50 polar interactions. The related plots are presented in Table S1. a) The calculated R-factor serves as an analogy to the residual minimized in crystallographic structure refinement. A desirable value of R-factor would be less than 0.1. b) The force field- based atomic charge parameters were scaled to yield correct net charge on each fragment system.

### Estimation of Errors in Protein Folds

With this database of interaction energy errors in place, lower-limit estimates of both systematic and random errors can be obtained for protein fold energies. Along with calculating the energy of a protein fold, an analysis of its interacting fragment components can be made. By determining the interaction type, an estimate for a fragment's contribution to the overall error can be retrieved from the database. The overall error is then propagated as
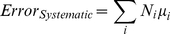
(2)

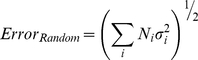
(3)where *i* runs over all interaction types (e.g. polar, nonpolar) stored in the database, *N_i_* is the number of interactions of type *i* found in the analyzed protein fold, and *μ_i_* and *σ_i_^2^* are the mean error per interaction and variance about the mean error for interaction type *i*. Note that total systematic error depends on the number of each type of interaction and thus will not exactly cancel when comparing different protein folds, since the folds may have different numbers of interaction types. The evaluated total systematic error should be subtracted from the evaluated energy and the evaluated total random error can be reported along with the corrected energy value.

In the case of B97-D/TZVP for ubiquitin (Figure 3), by using the appropriate error probability density functions we estimate the total systematic error to be 17.3 kcal/mol and the random error to be 8.9 kcal/mol. Hence, the estimated systematic error is comparable to a typical folding free energy of a protein, but this error can be corrected. Unfortunately, the remaining random error is still a significant portion of a typical folding free energy. Any other protein fold with a calculated energy within this 8.9 kcal/mol error bar should be considered indistinguishable from the native fold by the computational method. The B97-D/TZVP case represents a favorable example with small mean errors and relatively tight error distributions (see Table 1 for other examples), but it would be computationally intractable to use it to study hundreds or thousands of decoys for a system of the size of ubiquitin. More approximate and computationally accessible methods yield higher estimated errors. For example, ff99sb yields a systematic error of 66.0 kcal/mol and a random error of 18.4 kcal/mol. Such magnitudes of error bars are disturbing, since any non-native structure lying within the 18.4 kcal/mol range could not be distinguished from the native structure with ff99sb. Furthermore, these error bars become even larger as larger proteins with more molecular contacts are examined.

The magnitudes of these errors lead us to predict that current physics-based scoring functions used in *ab initio* protein folding studies can have total energy errors much larger than the folding free energies of typical proteins. Hence, we conclude that accurate energy computation and error reduction represents a major stumbling block along with sampling in the achievement of an ultimate solution to the *ab initio* protein-folding problem. However, we are now in a position to correct for systematic errors, thereby improving our computational outcomes.

### Discrimination between Native and Decoy folds – the Rosetta Decoy Set

In order to test our error hypothesis, we performed energy calculations and error corrections on a portion of the Rosetta decoy set, which contained 49 protein systems. Each one comprised a crystal structure from the Protein Databank, 20 versions of the crystal structure that were relaxed with the Rosetta score function, and 100 low energy decoys produced by Rosetta [55]. The protein structures ranged from 50 to 146 residues in chain length, so semiempirical QM calculations (utilizing modern linear scaling methods) were feasible. Energies of all 5929 protein structures were calculated with ff99sb, PM6, and PM6-DH2. The ff99sb calculations were performed with the generalized Born implicit solvent model, and the PM6 and PM6-DH2 calculations used the COSMO solvent model in MOPAC. Each structure was then analyzed for the number of nonpolar and polar interactions and the corresponding energy was corrected according to the appropriate error probability density functions. To measure improvement due to energy corrections, three values were monitored: EGAP, z-score, and EBO. The EGAP (energy gap) was defined as the difference between the energies of the lowest energy decoy and the lowest native structure. The z-score is the ratio of the difference between the lowest native fold energy and the average energy of all folds to the standard deviation of all fold energies. EBO (error bar overlap) yields true if the native structure is found to lie within the lowest energy error bar; otherwise it is false.

The results for the Rosetta decoy set analysis can be found in Tables S2, S3, S4, and will only be summarized here. By measuring EGAP, improvements due to error corrections were observed in 27, 36, and 31 of the protein datasets for ff99sb, PM6, and PM6-DH2, respectively. By measuring z-scores, improvements were seen in 38, 41, and 39 of the systems. The native structures were found within the lowest energy error bar in 45, 49, and 49 of the systems. Overall, systematic error correction offers some benefit, but improvement was not uniform. We observed that, while the magnitudes of energy corrections varied over a set of decoys, that variation was small. That is, both native and decoy folds had significant systematic errors, but the changes in relative energies after error correction were usually small. The decoy set for PDBID: 1H6Z, for example, had an average energy correction of 51.3±5.2 kcal/mol for ff99sb, and the native structure had an error correction of 56.9 kcal/mol. Although much of the systematic error canceled when measuring EGAP, the improvements due to error correction can still be significant compared to folding free energies. This would especially be true if we had included more non-native decoy folds in our analysis. The decoys in this set are very native-like and have roughly the same number of intramolecular contacts, leading to similar magnitudes of error corrections. The difference in systematic errors between a native structure and a partially unfolded structure is expected to be much greater.

**Figure 4:**
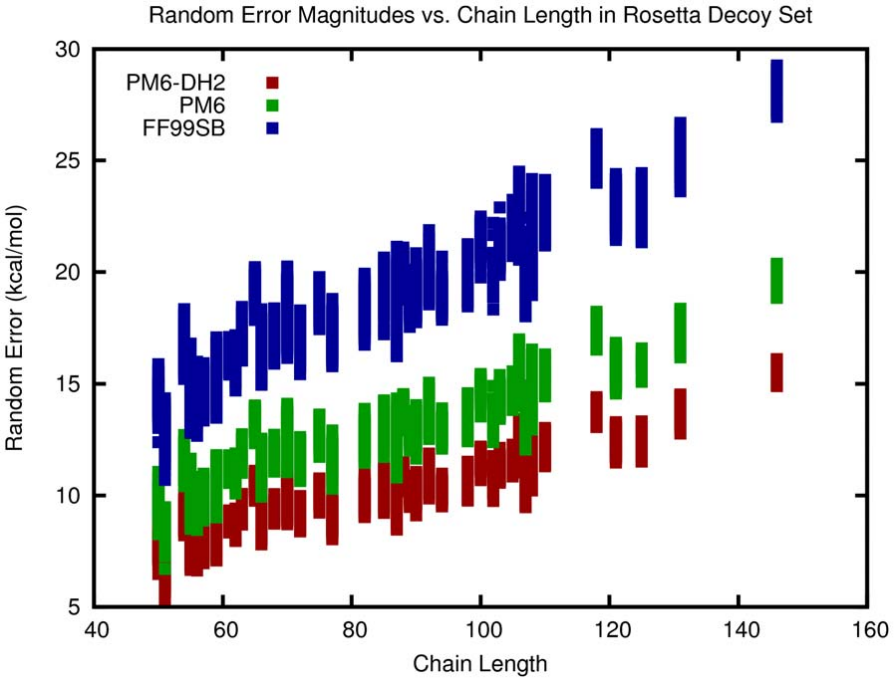
Dependence of random error estimates on chain length. Larger protein folds have more intramolecular interactions and thus larger propagated random errors in evaluated total energies. This effect is expected to lead to difficulty in predicting the native folds of large proteins since it leads to unpredictable distortions in the overall energy surface.

While systematic error can be estimated and removed, random error in energy scores isn't easily correctable and represents poor precision in scoring functions. After energy corrections, the native structure of 1H6Z was not the lowest energy structure with ff99sb, but its corrected energy was found to lie within the lowest energy error bar. This result highlights a main disadvantage of using a method with large random error since the native and decoy folds could not be distinguished due to overlapping random error bars. To highlight the dependence of the total random error on system size, we estimated the total random error of the 5929 protein folds with the three scoring functions. The relationship is shown in Figure 4. As we examine larger proteins, their potential energy surfaces should become increasingly more distorted due to increased random error in the energy functions, which can have unpredictable effects on the free energy landscape.

## Discussion

Folded proteins are characterized by numerous van der Waals and hydrogen bonding interactions that need to be accurately accounted for when using physics-based score functions. Even small errors in calculated energies between interacting partners within a protein quickly accumulate to produce large overall uncertainties in calculated total energies. This effect of error propagation distorts the calculated potential energy surface of a protein in a very complicated way, and therefore alters the shape of the folding funnel in ways that are difficult (if possible) to predict. One is only able to distinguish protein folds by energy when energy differences are larger than their individual error bars. Rather than having few native-like structures at the bottom of the folding funnel, it now should be extended to include any fold within the lowest energy error bar at the bottom. The bottom of the folding funnel may even be populated with non-native states predicted to be native by the scoring function, with the true native states higher in calculated energy but perhaps with error bars overlapping with the incorrectly predicted native states.

Since the free energy difference between a native and denatured protein fold can be on the order of 10–20 kcal/mol, the errors in interaction energies of the magnitude predicted herein suggest that we are a long way from computing energies between native and decoy folds at a level of accuracy necessary to generally solve the *ab initio* protein folding problem, especially as larger proteins are examined.

We have presented and demonstrated the use of a method to estimate the magnitude of errors in computed energies of proteins and shown that these can be corrected for in part, thereby improving results obtained from physics-based scoring functions. Systematic error correction can be applied as an endpoint calculation or it can be computed on the fly, for example, in interactive protein folding gaming exercises [56]. In addition, the generation of error probability density functions provides a straightforward method of analyzing and comparing different score functions in terms of their ability to accurately model molecular interactions. The research outlined herein brings a new level of sophistication to energy computation that has largely been lacking in computational biology and chemistry, opening the door for novel ways to compare and improve modern scoring functions used in studying complex systems with large numbers of inter- and intramolecular interactions.

## Supporting Information

Table S1 **Table of error probability density functions for each method studied.** The blue curves represent the error distributions of the nonpolar interactions, the red curves correspond to polar interactions, and the black curves represent all interactions. The numbers below each plot represent the expected systematic and random error in the composite ubiquitin system. Energy units are in kcal/mol.
(PDF)

Table S2 **Table displaying statistics related to the analysis of the Rosetta decoy set before and after energy corrections for the ff99sb force field.**
(PDF)

Table S3 **Table displaying statistics related to the analysis of the Rosetta decoy set before and after energy corrections for PM6.**
(PDF)

Table S4 **Table displaying statistics related to the analysis of the Rosetta decoy set before and after energy corrections for PM6-DH2.**
(PDF)
