# Supplementary figures and images for: The Energy Computation Paradox and ab initio Protein Folding
Source: PLoS One. 2011 Apr 25;6(4):e18868. doi: 10.1371/journal.pone.0018868 (PMC3081830; doi:10.1371/journal.pone.0018868)

Table S1: Error Probability Distributions

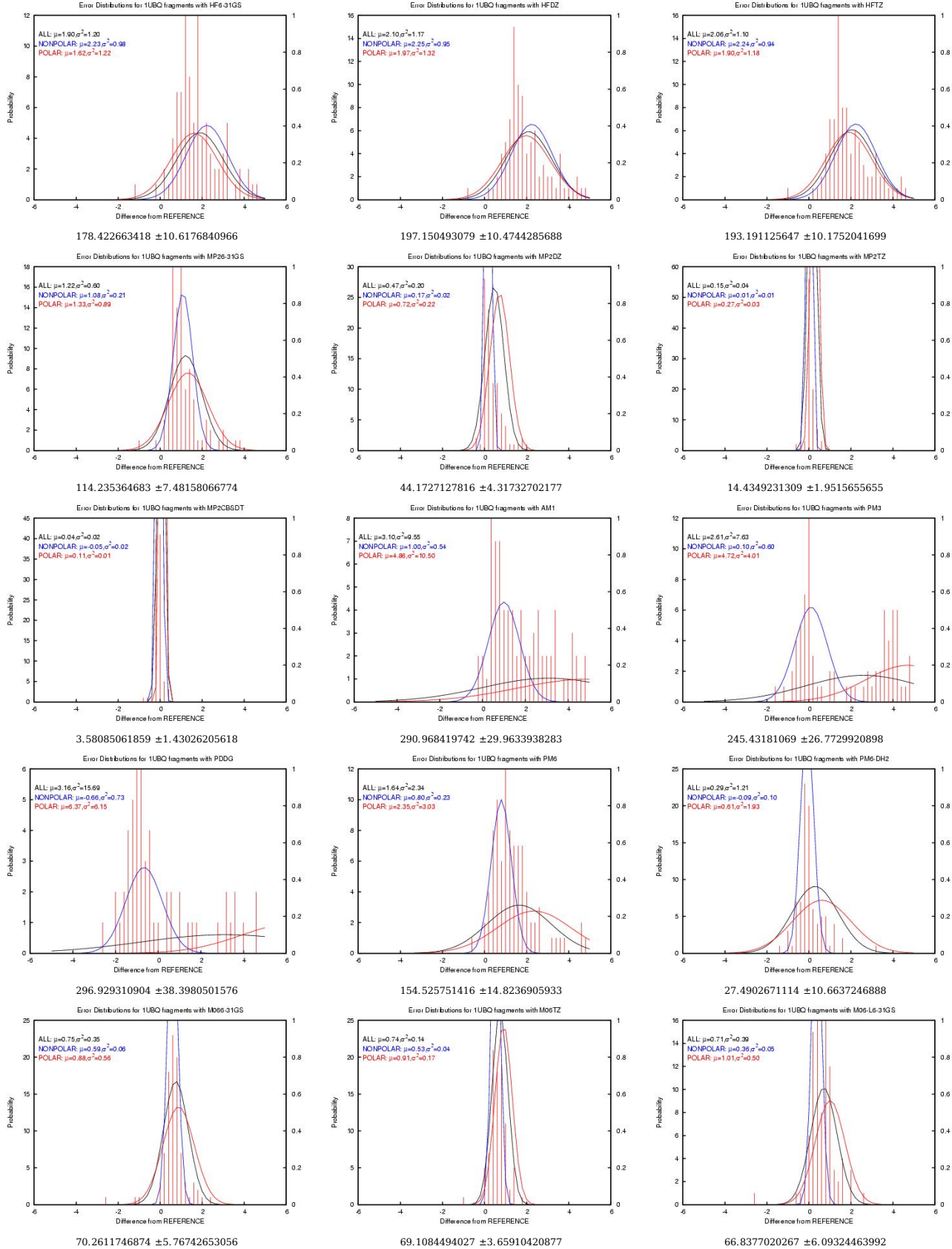

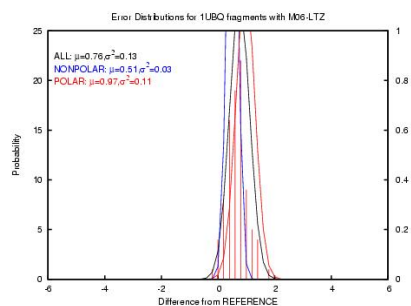

71.5254244335  $\pm$  3.446003017

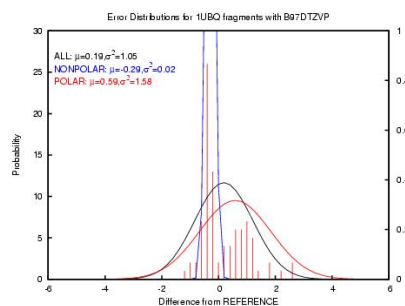

17.5449494975  $\pm$  9.94931169781

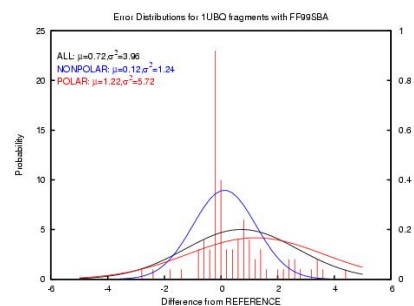

67.656221894  $\pm$  19.2986388823

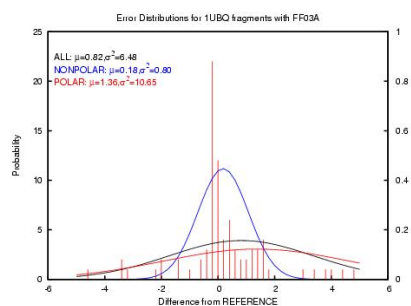

77.1363262418  $\pm$  24.673538514

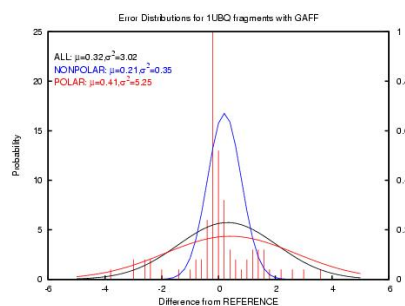

29.7562451997  $\pm$  16.8519087374

Supplement: Table S1 — Table of error probability density functions for each method studied. The blue curves represent the error distributions of the nonpolar interactions, the red curves correspond to polar interactions, and the black curves represent all interactions. The numbers below each plot represent the expected systematic and random error in the composite ubiquitin system. Energy units are in kcal/mol. (PDF) [file pone.0018868.s001.pdf]
