# Supplementary material for: The Energy Computation Paradox and ab initio Protein Folding
Source: PLoS One. 2011 Apr 25;6(4):e18868. doi: 10.1371/journal.pone.0018868 (PMC3081830; doi:10.1371/journal.pone.0018868)
Supplement: Table S2 — Table displaying statistics related to the analysis of the Rosetta decoy set before and after energy corrections for the ff99sb force field. (PDF) [file pone.0018868.s002.pdf]

Table S2: ff99sb

NAME	EGAPBEFORE	EGAPAFTER	IMPROVEMENT	ZSCOREBEFORE	ZSCOREAFTER	IMPROVEMENT	EBO
1a32	-8.2	-8.84	FALSE	-0.870404942	-1.113017656	TRUE	TRUE
1a68	-9.2	38.28	TRUE	-3.466960586	-3.674105168	TRUE	TRUE
1acf	20.1	28.58	TRUE	-1.251684007	-1.636689082	TRUE	TRUE
1ail	-18.3	-24.28	FALSE	-1.790384542	-1.545763233	FALSE	TRUE
1aiu	15	45.08	TRUE	-3.570643409	-3.799889304	TRUE	TRUE
1b3a	-7.1	-6.26	TRUE	-1.719537673	-1.876386378	TRUE	TRUE
1bgf	44.2	34.84	FALSE	-3.993805555	-3.556683803	FALSE	TRUE
1bk2	30.6	33.08	TRUE	-2.649073758	-2.936332053	TRUE	TRUE
1bkr	26.1	16.96	FALSE	-2.567031599	-2.597446314	TRUE	TRUE
1bq9	11	24.74	TRUE	-2.489955356	-2.781951428	TRUE	TRUE
1c8c	-17.4	-17.52	FALSE	-0.442263368	-0.491959382	TRUE	TRUE
1c9o	11.4	16.54	TRUE	-2.389257777	-2.573429946	TRUE	TRUE
1cc8	11.4	12.6	TRUE	-2.739383236	-2.725638122	FALSE	TRUE
1cei	36.9	34.4	FALSE	-3.942498757	-3.61053182	FALSE	TRUE
1ctf	22.1	32.2	TRUE	-1.374712557	-1.787035057	TRUE	TRUE
1dhn	4.5	49.88	TRUE	-3.502296512	-3.457848329	FALSE	TRUE
1e6i	-15.3	63.68	TRUE	-2.771875767	-2.923423532	TRUE	TRUE
1enh	2.2	0.66	FALSE	-2.204318014	-2.55866139	TRUE	TRUE
1ew4	4.7	14.42	TRUE	-0.1	-0.100503782	TRUE	TRUE
1eyv	-49.6	22.58	TRUE	-2.226197593	-2.548529972	TRUE	TRUE
1fkb	59.9	145.7	TRUE	-5.559315855	-6.094975988	TRUE	TRUE
1gvp	51.1	48.14	FALSE	-3.355233644	-4.319131367	TRUE	TRUE
1hz6	-23.6	-27.4	FALSE	-0.882708083	-0.654952918	FALSE	TRUE
1ig5	-14.2	-19.2	FALSE	-1.248003117	-1.33845356	TRUE	TRUE
1iib	-59.2	52	TRUE	-3.405973829	-4.504575722	TRUE	TRUE
1kpe	-4.3	-3.56	TRUE	-2.127416789	-2.208486182	TRUE	TRUE
1lou	64.4	70.24	TRUE	-3.587160286	-4.383439543	TRUE	TRUE
1opd	57	61.86	TRUE	-4.327881468	-4.739407289	TRUE	TRUE
1pgx	2.6	2.76	TRUE	-1.427141599	-1.765212833	TRUE	TRUE
1ptq	-14.9	-31.36	FALSE	-0.346760779	-0.064013871	FALSE	FALSE
1r69	-25.4	-37.24	FALSE	-1.13734593	-0.858683652	FALSE	FALSE
1scj	-20.9	-26.54	FALSE	-0.612075077	-0.758394288	TRUE	TRUE
1shf	27.1	33.92	TRUE	-2.148954878	-2.734314392	TRUE	TRUE
1ten	50.3	49.42	FALSE	-4.823522386	-4.771071705	FALSE	TRUE
1tig	-19.2	-25.68	FALSE	-0.748874711	-0.744274073	FALSE	TRUE
1tul	96.1	112.6	TRUE	-3.247048127	-5.898013348	TRUE	TRUE
1ugh	9.7	19.08	TRUE	-2.810717242	-3.070947064	TRUE	TRUE
1urn	31.4	36.38	TRUE	-3.298362837	-3.320131301	TRUE	TRUE
1utg	-13.1	-4.62	TRUE	-0.585224147	-1.868492891	TRUE	TRUE
1vcc	29	28.68	FALSE	-3.455357154	-3.603569812	TRUE	TRUE
1vie	48	57.52	TRUE	-4.184231442	-4.904069565	TRUE	TRUE
1vls	-81.5	-100.06	FALSE	0.286266757	1.10510155	FALSE	FALSE
1who	71.3	86.84	TRUE	-4.634861079	-4.899850231	TRUE	TRUE
256b	13.6	10.18	FALSE	-1.841641915	-2.106254288	TRUE	TRUE
2acy	102.4	93.24	FALSE	-4.680072769	-5.191250537	TRUE	TRUE
2ci2	-33.2	-32.84	TRUE	-0.222462487	-0.261423084	TRUE	FALSE
2tif	25	22.3	FALSE	-1.41343928	-1.50730499	TRUE	TRUE
4ubp	-10	-15.52	FALSE	-1.20903077	-1.780567411	TRUE	TRUE
5cro	3.3	1.46	FALSE	-0.7577955	-2.299394193	TRUE	TRUE
NUMBER TRUE			27			38	45
RATIO			0.551020408			0.775510204	0.918367347

EGAP=E(lowest decoy)-E(native)

Zscore=[E(native)-E(mean)]/sigma

EBO=TRUE if native error bar within lowest error bar
