# Supplementary material for: The Energy Computation Paradox and ab initio Protein Folding
Source: PLoS One. 2011 Apr 25;6(4):e18868. doi: 10.1371/journal.pone.0018868 (PMC3081830; doi:10.1371/journal.pone.0018868)
Supplement: Table S3 — Table displaying statistics related to the analysis of the Rosetta decoy set before and after energy corrections for PM6. (PDF) [file pone.0018868.s003.pdf]

Table S3: PM6

NAME	EGAPBEFORE	EGAPAFTER	IMPROVEMENT	ZSCOREBEFORE	ZSCOREAFTER	IMPROVEMENT	EBO
1a32	107.81003	110.99003	TRUE	-6.776229849	-5.972945028	FALSE	TRUE
1a68	62.40122	211.23871	TRUE	-5.524857362	-6.047920021	TRUE	TRUE
1acf	132.48612	139.441	TRUE	-2.681814288	-4.447706011	TRUE	TRUE
1ail	112.75773	91.21773	FALSE	-4.360501828	-4.036603275	FALSE	TRUE
1aiu	179.23289	209.71576	TRUE	-6.031612583	-6.020053123	FALSE	TRUE
1b3a	99.73842	92.56842	FALSE	-5.41064807	-5.395253597	FALSE	TRUE
1bgf	141.82308	140.71196	FALSE	-5.449061084	-5.488790965	TRUE	TRUE
1bk2	72.57224	80.38757	TRUE	-4.810722535	-5.239049916	TRUE	TRUE
1bkr	111.34915	130.63915	TRUE	-4.603217788	-4.755533012	TRUE	TRUE
1bq9	105.05331	126.36346	TRUE	-5.494595187	-5.802103967	TRUE	TRUE
1c8c	86.84539	82.87539	FALSE	-3.931114144	-5.579214423	TRUE	TRUE
1c9o	113.17865	111.57865	FALSE	-5.574807096	-5.675161057	TRUE	TRUE
1cc8	109.7024	113.7024	TRUE	-5.391264053	-5.56909359	TRUE	TRUE
1cei	206.04689	221.23689	TRUE	-5.988100209	-6.133124405	TRUE	TRUE
1ctf	120.29389	141.01389	TRUE	-3.630934581	-5.967446178	TRUE	TRUE
1dhn	112.89637	230.7799	TRUE	-5.260159841	-5.539250747	TRUE	TRUE
1e6i	170.28449	267.16184	TRUE	-6.460567412	-6.650998237	TRUE	TRUE
1enh	89.32891	94.9859	TRUE	-5.536605626	-5.656904077	TRUE	TRUE
1ew4	108.28673	171.75449	TRUE	-4.679757132	-5.488917586	TRUE	TRUE
1eyv	81.40205	214.97838	TRUE	-4.919297883	-5.407486289	TRUE	TRUE
1fkb	189.89786	200.28786	TRUE	-5.56997807	-5.47560342	FALSE	TRUE
1gvp	141.92979	142.86951	TRUE	-4.539635857	-5.560040855	TRUE	TRUE
1hz6	29.0331	79.73148	TRUE	-5.720113983	-6.497342765	TRUE	TRUE
1ig5	91.35263	85.87025	FALSE	-4.233588899	-4.666742589	TRUE	TRUE
1iib	27.2809	179.44318	TRUE	-5.788295552	-7.019805486	TRUE	TRUE
1kpe	154.81529	168.38529	TRUE	-5.700115156	-5.797136222	TRUE	TRUE
1lou	135.56697	169.91697	TRUE	-5.037909284	-5.349156585	TRUE	TRUE
1opd	148.43156	163.36422	TRUE	-5.653760375	-6.450020371	TRUE	TRUE
1pgx	103.1054	96.97566	FALSE	-5.282541827	-7.087117353	TRUE	TRUE
1ptq	51.33102	24.26102	FALSE	-2.967013948	-3.125790941	TRUE	TRUE
1r69	95.45322	76.35322	FALSE	-4.031645302	-5.564867093	TRUE	TRUE
1scj	83.97225	103.09704	TRUE	-3.761527859	-4.525150033	TRUE	TRUE
1shf	137.7655	148.62895	TRUE	-4.952202731	-5.947941756	TRUE	TRUE
1ten	143.4062	159.3762	TRUE	-6.329045979	-6.293088153	FALSE	TRUE
1tig	104.61389	107.16625	TRUE	-4.816607887	-4.629704877	FALSE	TRUE
1tul	156.13817	219.96817	TRUE	-4.536247971	-6.526901544	TRUE	TRUE
1ugh	166.77598	181.46008	TRUE	-6.136374959	-6.285115588	TRUE	TRUE
1urn	136.43571	154.85095	TRUE	-5.142384095	-5.237541851	TRUE	TRUE
1utg	127.31781	127.59907	TRUE	-4.498317815	-6.025056193	TRUE	TRUE
1vcc	136.73834	153.10537	TRUE	-5.406172333	-5.569788447	TRUE	TRUE
1vie	113.96711	125.11711	TRUE	-5.255790851	-5.870919525	TRUE	TRUE
1vls	102.99749	94.96815	FALSE	-3.968772499	-4.28096723	TRUE	TRUE
1who	178.1494	212.27527	TRUE	-6.539987285	-6.888049762	TRUE	TRUE
256b	212.85971	209.13141	FALSE	-7.481795572	-7.232659051	FALSE	TRUE
2acy	165.17975	205.91975	TRUE	-5.458834384	-6.210373397	TRUE	TRUE
2ci2	69.95746	82.74746	TRUE	-4.067506853	-4.987526972	TRUE	TRUE
2tif	25.11465	33.66098	TRUE	-2.842837888	-3.023525225	TRUE	TRUE
4ubp	160.08663	156.14663	FALSE	-3.610762188	-5.323928095	TRUE	TRUE
5cro	103.52432	89.96432	FALSE	-2.818697322	-5.917061733	TRUE	TRUE
NUMBER TRUE			36			41	49
RATIO			0.734693878			0.836734694	1

EGAP=E(lowest decoy)-E(native)

Zscore=[E(native)-E(mean)]/sigma

EBO=TRUE if native error bar within lowest error bar
