# Supplementary material for: The Energy Computation Paradox and ab initio Protein Folding
Source: PLoS One. 2011 Apr 25;6(4):e18868. doi: 10.1371/journal.pone.0018868 (PMC3081830; doi:10.1371/journal.pone.0018868)
Supplement: Table S4 — Table displaying statistics related to the analysis of the Rosetta decoy set before and after energy corrections for PM6-DH2. (PDF) [file pone.0018868.s004.pdf]

Table S4: PM6-DH2

NAME	EGAPBEFORE	EGAPAFTER	IMPROVEMENT	ZSCOREBEFORE	ZSCOREAFTER	IMPROVEMENT	EBO
1a32	112.03431	111.50431	FALSE	-6.561201387	-6.355470837	FALSE	TRUE
1a68	60.89331	258.40545	TRUE	-6.033752979	-6.796052199	TRUE	TRUE
1acf	224.40972	218.03972	FALSE	-4.073181427	-5.677933851	TRUE	TRUE
1ail	90.14602	108.58507	TRUE	-4.470633728	-4.566861689	TRUE	TRUE
1aiu	202.09342	231.09252	TRUE	-6.4854251	-6.55254116	TRUE	TRUE
1b3a	92.92398	90.78398	FALSE	-6.026898762	-6.054796275	TRUE	TRUE
1bgf	228.2013	219.9213	FALSE	-7.200521534	-7.057990075	FALSE	TRUE
1bk2	102.63713	104.66713	TRUE	-6.19691702	-6.269296069	TRUE	TRUE
1bkr	201.31721	191.14721	FALSE	-6.090339335	-5.903682404	FALSE	TRUE
1bq9	140.93208	140.63208	FALSE	-6.685899659	-6.639248004	FALSE	TRUE
1c8c	90.29937	93.21937	TRUE	-4.220914544	-6.195364046	TRUE	TRUE
1c9o	127.84471	130.51471	TRUE	-6.137229006	-6.188338765	TRUE	TRUE
1cc8	149.24391	150.55391	TRUE	-6.375566673	-6.352878198	FALSE	TRUE
1cei	220.89953	218.39953	FALSE	-6.457702636	-6.416078151	FALSE	TRUE
1ctf	147.58693	147.94693	TRUE	-4.141243416	-6.354046294	TRUE	TRUE
1dhn	94.7515	280.58882	TRUE	-5.80573848	-6.327695135	TRUE	TRUE
1e6i	153.61834	276.02521	TRUE	-6.639885017	-7.08294095	TRUE	TRUE
1enh	95.4361	93.77651	FALSE	-5.989954519	-5.944982192	FALSE	TRUE
1ew4	96.89932	258.6563	TRUE	-6.471090405	-7.260455522	TRUE	TRUE
1eyv	108.10039	248.21697	TRUE	-5.947496494	-6.350135196	TRUE	TRUE
1fkb	323.35459	326.24459	TRUE	-7.097431143	-7.152441048	TRUE	TRUE
1gvp	142.98415	143.24415	TRUE	-5.275682134	-6.619290422	TRUE	TRUE
1hz6	10.21568	94.02833	TRUE	-5.774103049	-6.792614265	TRUE	TRUE
1ig5	98.28397	93.85397	FALSE	-4.439882045	-4.839562711	TRUE	TRUE
1iib	57.61356	182.29637	TRUE	-6.434634273	-7.399594693	TRUE	TRUE
1kpe	133.86449	229.44476	TRUE	-6.429467212	-6.877584493	TRUE	TRUE
1lou	208.89225	210.30225	TRUE	-6.393145471	-6.307769721	FALSE	TRUE
1opd	187.00644	190.16644	TRUE	-6.603268771	-7.072399468	TRUE	TRUE
1pgx	97.78714	97.70714	FALSE	-5.643542158	-7.217044941	TRUE	TRUE
1ptq	42.40434	33.89434	FALSE	-2.719899959	-3.652902867	TRUE	TRUE
1r69	107.94074	101.82074	FALSE	-4.152411154	-6.204908377	TRUE	TRUE
1scj	118.71535	120.64654	TRUE	-4.634588684	-5.463040466	TRUE	TRUE
1shf	173.11324	176.47324	TRUE	-5.906111001	-6.634773272	TRUE	TRUE
1ten	217.61004	228.69899	TRUE	-7.479290562	-7.514665536	TRUE	TRUE
1tig	82.74895	121.28535	TRUE	-5.225814051	-5.356179406	TRUE	TRUE
1tul	247.92564	255.85564	TRUE	-6.302592249	-7.313244758	TRUE	TRUE
1ugh	146.98995	226.12669	TRUE	-6.701172401	-7.015129279	TRUE	TRUE
1urn	122.83494	184.91935	TRUE	-6.266904419	-6.47270579	TRUE	TRUE
1utg	119.91854	120.89854	TRUE	-4.325981532	-6.382829786	TRUE	TRUE
1vcc	129.31218	160.58405	TRUE	-5.685903278	-5.747201912	TRUE	TRUE
1vie	139.17166	143.78166	TRUE	-6.20964744	-6.451630006	TRUE	TRUE
1vls	139.49141	134.13141	FALSE	-4.868862101	-5.263478033	TRUE	TRUE
1who	225.82966	235.18186	TRUE	-7.325413695	-7.370856149	TRUE	TRUE
256b	217.06086	222.23086	TRUE	-7.570281422	-7.644571634	TRUE	TRUE
2acy	252.84352	242.82352	FALSE	-7.422051497	-7.307882961	FALSE	TRUE
2ci2	87.62534	87.33534	FALSE	-4.802805192	-5.372325462	TRUE	TRUE
2tif	56.24247	53.22247	FALSE	-3.596695667	-3.507714152	FALSE	TRUE
4ubp	189.71395	189.53395	FALSE	-3.78775437	-5.555442941	TRUE	TRUE
5cro	88.94259	87.70259	FALSE	-2.614394915	-6.090272627	TRUE	TRUE
NUMBER TRUE			31			39	49
RATIO			0.632653061			0.795918367	1

EGAP=E(lowest decoy)-E(native)

Zscore=[E(native)-E(mean)]/sigma

EBO=TRUE if native error bar within lowest error bar
